# Evaluation of the Condyle-Fossa Relationship in Angle’s Class II Division 1 Malocclusions Using Cone-Beam Computed Tomography: A Descriptive Cross-Sectional Study in Humans

**DOI:** 10.7759/cureus.89202

**Published:** 2025-08-01

**Authors:** Pappala Venkata Prasanna, RSVM Raghu Ram, Soorabathula Sonika Mani kiran, Desu Venkata Naga Durga Harshini, Neelapala Rohini, Inuganti Ranganayakulu

**Affiliations:** 1 Orthodontics and Dentofacial Orthopaedics, GSL Dental College & Hospital, Rajahmundry, IND; 2 Orthodontics, GSL Dental College & Hospital, Rajahmundry, IND

**Keywords:** cbct, class ii division 1, condyle-fossa relationship, cone-beam computed tomography, subdivision, temporomandibular joint spaces

## Abstract

Background

The condyle-fossa relationship is essential for smooth, pain-free jaw movement, relying on symmetrical and balanced condyles. However, this relationship may vary in individuals with malocclusions. Despite its clinical significance, few studies have examined the three-dimensional aspects of condyle morphology and position in class II division 1 malocclusion, and there is a lack of comprehensive data on its subdivision.

Aim and objective

This study aimed to assess and compare the condyle-fossa relationship, condyle positions, and the dimensional and positional symmetry between the right and left condyles in class II division 1 malocclusion and its subdivisions.

Methodology

Eighty patients aged 18 years and older were categorized into two groups: the control group (class II division 1) and the test group (class II division 1, subdivision), with 40 individuals in each group. Cone-beam computed tomography (CBCT) images were used for the evaluation, employing Will Master software (HDX WILL Corporation, Korea). Digital measurements, including condyle-fossa depth, joint spaces, and positional parameters, were taken using OnDemand 3D software (version 2016.12, Cybermed, Korea). Statistical analysis included descriptive statistics, t-tests, and one-way analysis of variance (ANOVA), with Tukey’s post hoc tests for multiple pairwise comparisons.

Results

No significant differences were found between the right and left sides across all parameters in the control group. In contrast, the test group showed significant differences in condylar fossa depth (P=0.001) and superior joint space (P=0.006). The class I side exhibited a higher mean condylar fossa depth than the class II side, while the class II side displayed a higher mean superior joint space. Comparisons between the control and test groups revealed significant differences in mean condylar fossa depth (P=0.001), anterior joint space (P=0.014), and mediolateral diameter of the condylar process (P=0.009).

Conclusions

Significant differences were observed in condylar fossa depth and superior joint space between the class II division 1 subdivision group and the class II division 1 group. Additionally, asymmetry in condylar positions was noted, with the class I side showing a deeper condylar fossa and the class II side displaying a larger superior joint space.

## Introduction

A harmonious relationship between occlusion and the morphological positioning of the temporomandibular joint (TMJ) apparatus is pivotal for the condyle's smooth functioning, which is also influenced by the joint capsule and articular disc. The TMJ's morphological characteristics, including the condyle-fossa relationship, condyle concentricity, and symmetry between the right and left condyles, are critical determinants of its health and function. The condyle-fossa relationship governs the movement of the condyle within the joint during jaw movements, ensuring pain-free and smooth articulation. Symmetrical dimensions and concentricity of the condyles within their respective fossae are essential for maintaining proper functional balance [1,2]. Martins et al. reported the mean joint space values as 1.86 mm anteriorly, 2.36 mm superiorly, and 2.22 mm posteriorly, with no significant differences between the right and left sides [3]. Alterations in either occlusion or condyle position may influence each other, although the precise nature of this relationship is not yet fully understood.

Morphological changes and concentricity of the condyles in the mandibular fossa show a significant association with malocclusions. In hyperdivergent groups, the mandibular fossa is positioned more superiorly and anteriorly, while in hypo-divergent groups, it is more inferiorly and posteriorly located [4]. These positional changes are often accompanied by variations in vertical condyle inclination, condylar width, and length [5]. The glenoid fossa is positioned more posteriorly and distally in class II malocclusions compared to patients with class I malocclusion [6]. Condyles are most anteriorly positioned in skeletal class II, posteriorly in skeletal class I, and superiorly in skeletal class III malocclusions [7]. Class III individuals exhibit increased condylar width, height, and volume, along with a reduced superior joint space, while class II individuals demonstrate a reduced anterior joint space and increased posterior joint space [8]. Class II division 1 malocclusion is associated with a non-concentric condylar position, with the condyles being more anteriorly placed [9]. However, contrary results were reported by Gianelly et al., who found that class II malocclusions had concentrically positioned condyles [10].

Temporomandibular disorders (TMDs) are commonly associated with asymmetric condyles, their positions, and malocclusions. Marchesi et al. found a significant association between TMDs and class I, II, and III malocclusions with deep bite, open bite, and midline deviation [11]. Yanez-Vico et al. observed that shorter condylar height was a common feature in TMD patients compared with asymptomatic individuals [12]. The morphological changes and position of the TMJ are best evaluated using various diagnostic imaging techniques. Advanced radiological methods such as computed tomography (CT), magnetic resonance imaging (MRI), and cone-beam computed tomography (CBCT) are frequently used [13-15]. CBCT is a cutting-edge technology that is rapidly advancing in the field of dentistry. It allows for the clear visualization of areas of interest with high accuracy, without superimposition, in a three-dimensional (3D) view. While CT scans offer similar visualization, the advantages of CBCT include faster scan times, lower radiation exposure, compact size, and cost-effectiveness when compared with CT and MRI scans [16].

Currently, few studies document the 3D aspects of condyle morphology and position in class II division 1 malocclusion, with insufficient data on its subdivision. The findings from such studies could pave the way for more precise treatment planning for these malocclusions. The purpose of this study is to evaluate the condyle-fossa relationship, the concentric position of the condyles, and the dimensional and positional symmetry between the right and left condyles in class II division 1 and class II division 1 subdivision malocclusions using CBCT imaging. The present study sets the null hypotheses (H₀) as no significant difference in the condyle-fossa relationship, concentric positioning, or symmetry between class II division 1 and class II division 1 subdivision malocclusions. This provides the foundation for statistical testing and allows for evaluation of the research questions.

## Materials and methods

The descriptive cross-sectional human study was designed by the Strengthening the Reporting of Observational Studies in Epidemiology (STROBE) statement and complied with the Declaration of Helsinki guidelines for research involving human subjects. The study protocol was approved by the Institutional Ethics Committee of GSL Dental College and Hospital (GSLDC/IEC/2022/005). All patients whose records were used in this study received detailed information and provided informed consent for their participation.

To compare the quantitative study variables between class II, division 1 and class II, division 1 subdivision groups, the sample size was calculated using G*power software (ver 3.1.9.7, Heinrich-Heine-Universität Düsseldorf, Düsseldorf, Germany); with the parameters like independent samples t-test effect size - 0.7 based on Cohen’s large effect size assumption; α error probability - 0.05; power - 0.8; allocation ratio - 1:1.

A sample size of 68 was obtained using the aforementioned parameters, with 34 subjects in each of the two groups. The required sample size was rounded off to 40 in each group. A sample of 80 patients seeking orthodontic treatment and reporting to the Department of Orthodontics at GSL Dental College and Hospital, Rajahmundry, Andhra Pradesh, India, from August 2022 to September 2023, was considered. The sample was divided into two groups of 40 subjects, each with class II, division 1 and class II, division 1, subdivision malocclusions, respectively, and whose CBCT records were taken as part of the necessary radiographs.

The inclusion criteria of the patients were: (1) Angle’s class II, division 1 malocclusion; (2) Angle’s class II, division 1, subdivision malocclusion; (3) straight to a mild convex profile; (4) clinical Frankfort Mandibular Angle - average growth pattern; (5) age above 18 years; (6) full permanent dentition; and (7) aligned arches with mild acceptable anterior crowding ≤4 mm.

Exclusion criteria were as follows: (1) ongoing or previously done orthodontic treatment; (2) temporomandibular joint disorders; (3) history of temporomandibular trauma; (4) transverse discrepancies; (5) para-functional habits; (6) one-sided chewing habit; (7) craniofacial deformities; and (8) systemic disorders.

The individuals who met the inclusion criteria were informed about the research, and their CBCT scans were taken using CBCT equipment DENTRI (HDX WILL Corporation, Korea), and the image was reconstructed using Will Master software (WillCeph digital imaging software, version 1.0.0; HDX Will Corp, Korea). The scanned CBCT data were saved as Digital Imaging and Communications in Medicine (DICOM) files. The 3D images were reconstructed into multiplanar reformatted images (MPR) from the DICOM files using ON-DEMAND 3D software (version 2016.12, Cybermed, Korea).

**Figure 1:**
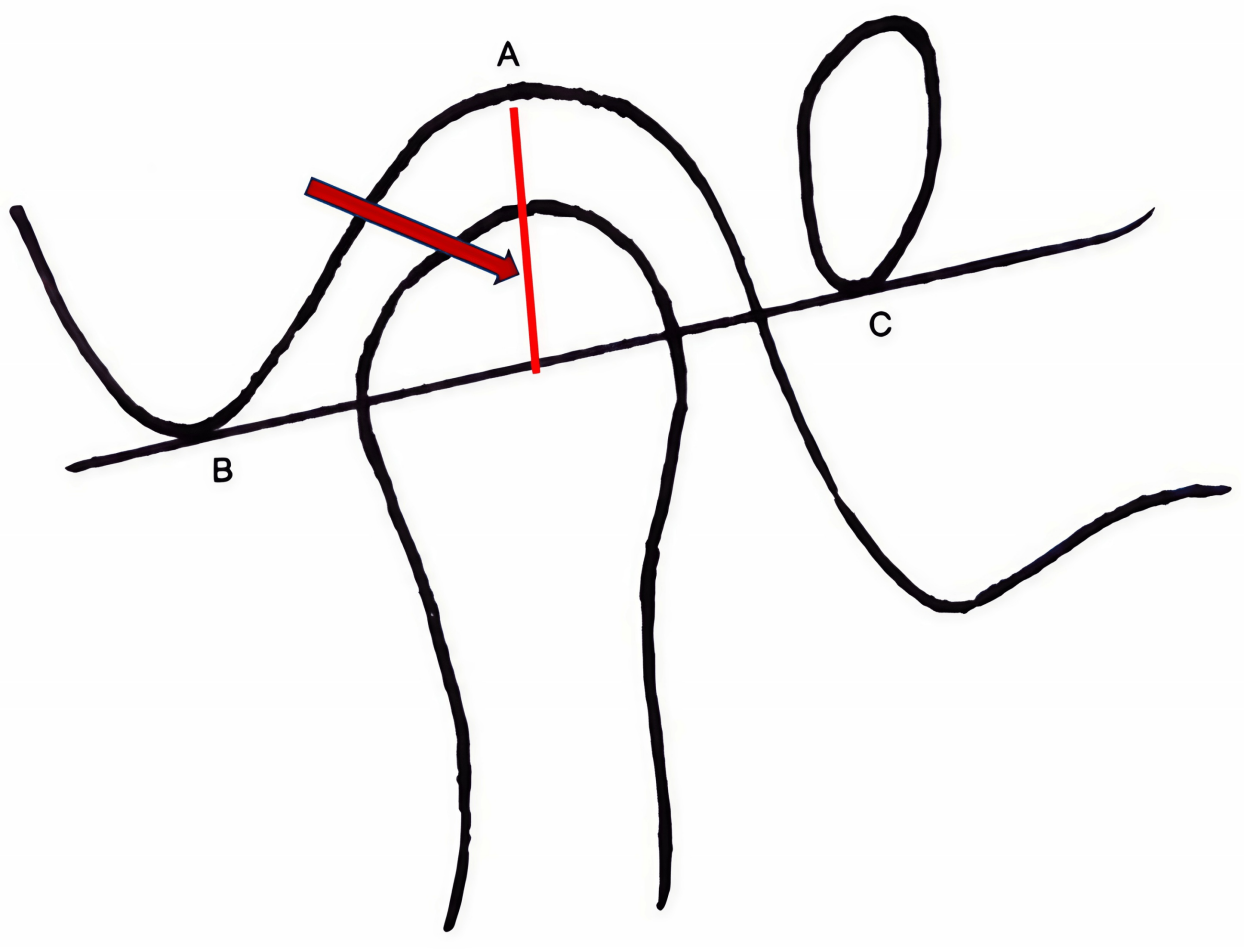
Diagrammatic representation of the depth of the mandibular fossa The distance is measured from the fossa's highest point (A) to the articular tubercle's (B) and auditory meatus's (C) lowest points.

**Figure 2:**
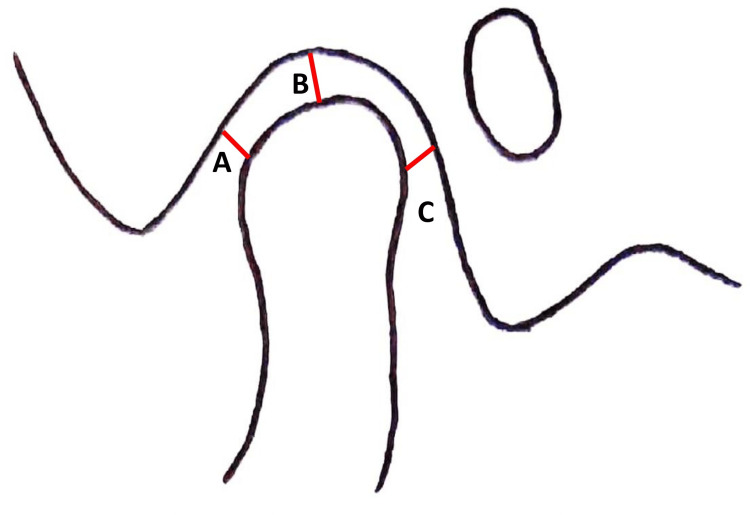
Diagrammatic representation of the joint spaces (A) anterior joint space; (B) superior joint space; (C) posterior joint space

The generated images were used for the assessment of the depth of the mandibular fossa on both sides, anterior, superior, and posterior joint spaces, and measurement of the parameters like greatest anteroposterior diameter, the mediolateral diameter of the condylar processes, the angle between geometric centers of condylar processes and mid-sagittal plane, and the anteroposterior difference between the geometric center of right and left condylar processes. The following measurements were evaluated on the sagittal plane: (1) Depth of mandibular fossa: The distance is determined by measuring from the utmost elevation of the fossa to the level established by the lowest points of both the articular tubercle and the auditory meatus (Figure 1); 

(2) Anterior joint space: It is the shortest distance between the most anterior point of the condyle and the posterior wall of the articular tubercle; (3) Superior joint space: It is the shortest distance between the most superior point of the condyle and the most superior point of the mandibular fossa; and (4) Posterior joint space: It is the shortest distance between the most posterior point of the condyle and the posterior wall of the mandibular fossa (Figure 2).

**Figure 3:**
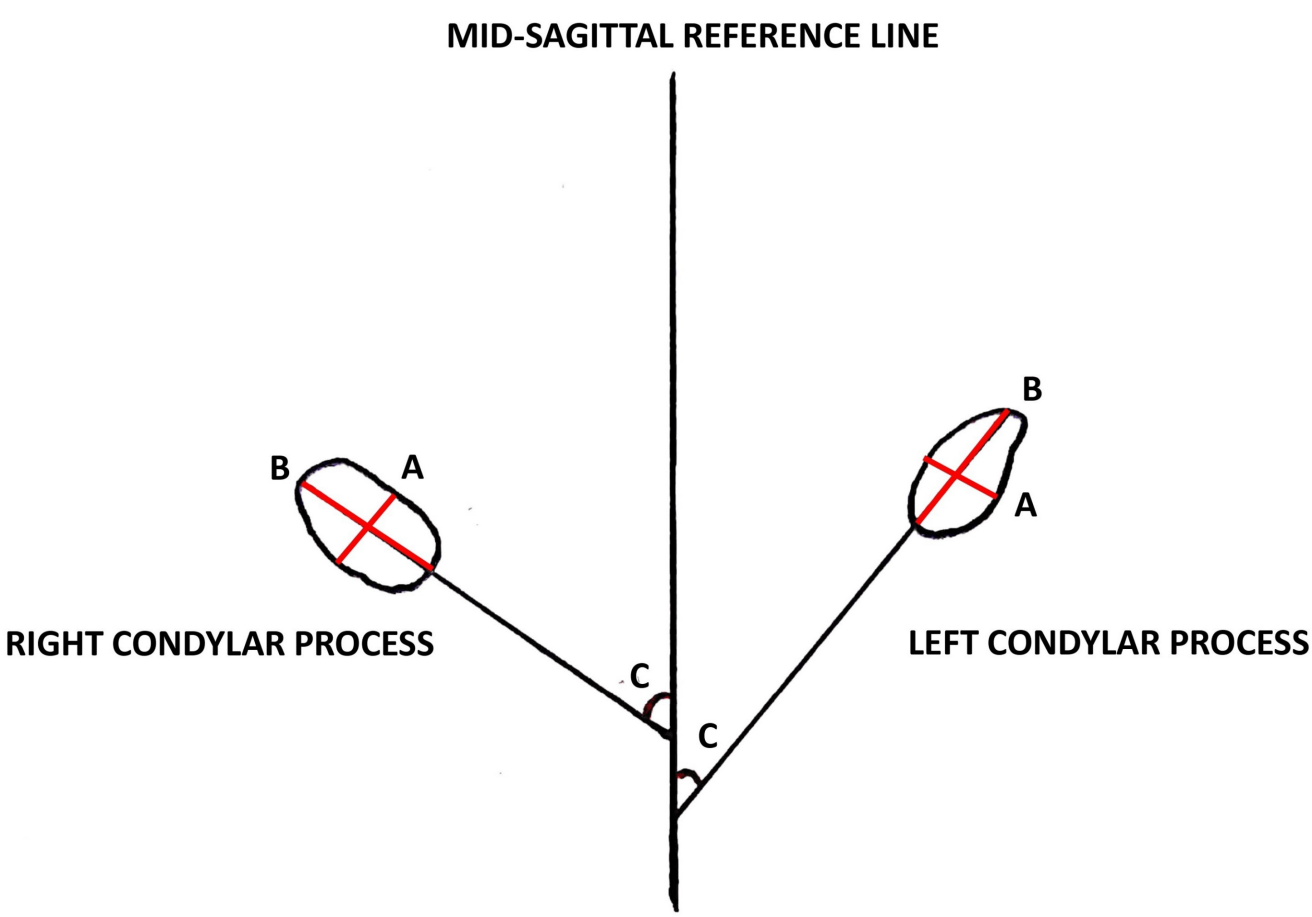
Line diagram representing the condyle in the mid-sagittal reference plane (A) The greatest anteroposterior diameter of the mandibular condylar process; (B) The greatest mediolateral diameter of the mandibular condylar process; (C) The lateromedial plane angle of the condylar process/mid-sagittal plane

**Figure 4:**
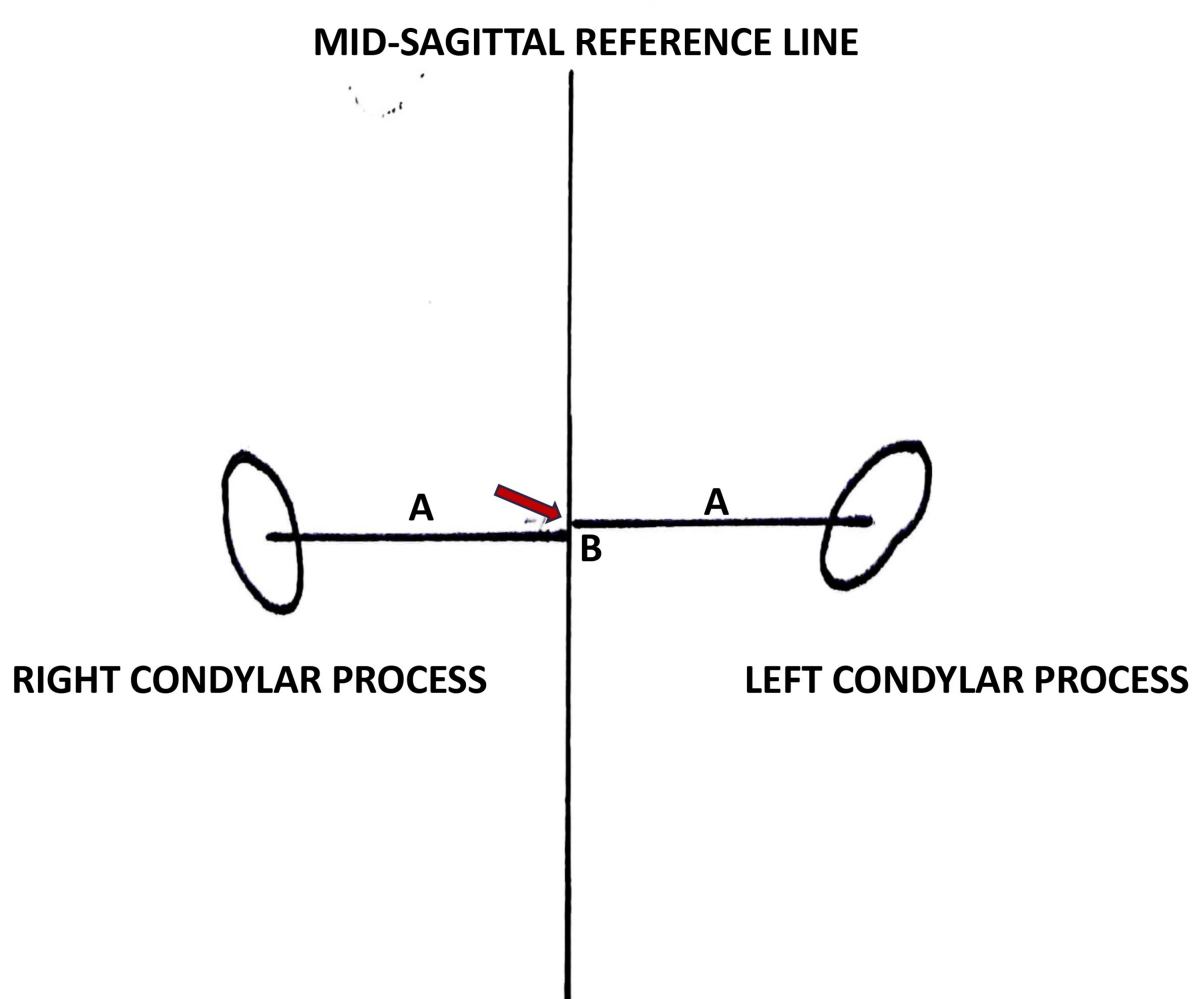
Line diagram representing the condyle in the mid-sagittal reference plane (A) The distance between the geometric center of the condylar processes to the mid-sagittal plane and (B) The anteroposterior difference of the condylar processes

The following parameters were measured on the axial plane: (1) The greatest anteroposterior diameter of the mandibular condylar processes; (2) The maximum width of the mandibular condylar processes in the mediolateral direction; (3) The angle between the long axis of the mandibular condylar processes and the mid-sagittal plane (Figure 3); 

(4) The distance between the geometric centers of the condylar processes and the mid-sagittal plane; and (5) The anteroposterior difference between the geometric center of the right and left condylar processes (Figure 4).

Measurements of the anterior and posterior joint spaces are compared for the right and left sides to assess the centric position of the condyles in the mandibular fossa in both groups. These measurements were observed and all were re-examined by the same researcher after two weeks and an intra examiner reliability analysis was conducted. The obtained data was statistically analyzed using IBM SPSS Statistics for Windows, Version 20 (Released 2011; IBM Corp., Armonk, New York, United States). Descriptive statistics, t-tests, and one-way analysis of variance, followed by Tukey’s post hoc tests for multiple pairwise comparisons, were employed to scrutinize the study data. P≤0.05 was considered statistically significant for all the employed tests. 

## Results

Age comparison

The control group had a mean age of 23.67±3.56 years, while the test group had a mean age of 23.82±3.75 years. There was no statistically significant difference in mean age between the groups (Table 1).

**Table 1:** Comparison of mean age between the study groups Unpaired t-test; p≤0.05 considered statistically significant

Group	Sample size (N)	Mean	Standard deviation	Standard error	P value
Control	40	23.67	3.56	0.56	0.854
Test	40	23.82	3.75	0.59	

Gender distribution

No significant differences in gender distribution was observed between the two groups. In the control group, 67.5% (n=27) were female patients, and 32.5% (n=13) were male patients. Similarly, the test group consisted of 60% (n=24) female participants and 40% (n=16) male participants.

**Table 2:** Comparison of gender distribution between the study groups Chi square test; p≤0.05 considered statistically significant

Group	Sample size ( N)	Male participants (%)	Female participants (%)	P value
Control	40	13 (32.5)	27 (67.5)	0.485
Test	40	16 (40)	24 (60)	

Descriptive statistics

Descriptive statistics for the study parameters in the Angle’s class II, division 1 (control) group, and for the Angle’s class II, division 1 subdivision (test) group are presented in Table 3 and Table 4, respectively.

**Table 3:** Descriptive statistics for the study parameters in the Angle’s class II, division 1 (control group)

Parameter	Side	Sample size (N)	Mean	Standard deviation	Standard error mean	95% confidence interval lower bound	95% confidence interval upper bound
Condylar fossa depth	Right	40	8.6335	0.78491	0.12411	8.390244	8.876756
	Left	40	8.4327	0.71958	0.11378	8.209691	8.655709
Anterior joint space	Right	40	2.8713	0.47263	0.07473	2.724829	3.017771
	Left	40	2.7083	0.43156	0.06823	2.574569	2.842031
Superior joint space	Right	40	2.5913	0.59117	0.09347	2.408099	2.774501
	Left	40	2.6648	0.62784	0.09927	2.470231	2.859369
Posterior joint space	Right	40	2.5685	0.5547	0.08771	2.396588	2.740412
	Left	40	2.4565	0.61515	0.09726	2.26587	2.64713
Anteroposterior diameter of the condylar process	Right	40	7.8915	1.0384	0.16419	7.569688	8.213312
	Left	40	7.9545	0.94533	0.14947	7.661539	8.247461
Mediolateral diameter of the condylar process	Right	40	19.7812	1.0835	0.17132	19.44541	20.11699
	Left	40	20.1337	1.15207	0.18216	19.77667	20.49073
Angle between the condylar long axis & mid-sagittal plane	Right	40	65.475	3.84453	0.60787	64.28357	66.66643
	Left	40	64.855	8.41098	1.32989	62.24842	67.46158
Distance between the geometric center of the condylar process & the mid-sagittal plane	Right	40	50.9605	3.38447	0.53513	49.91165	52.00935
	Left	40	51.8608	3.06744	0.48501	50.91018	52.81142
Anteroposterior difference between the right & left		40	0.7385	0.54639	.08639	0.569176	0.907824

**Table 4:** Descriptive statistics for the study parameters in the Angle’s class II, division 1, subdivision (test) group

Parameter	Side	Sample size (N)	Mean	Standard deviation	Standard error mean	95% confidence interval lower bound	95% confidence interval upper bound
Condylar fossa depth	Right	40	8.4565	0.76246	0.12056	8.220202	8.692798
	Left	40	7.8527	0.89018	0.14075	7.57683	8.12857
Anterior joint space	Right	40	2.5863	0.51328	0.08116	2.427226	2.745374
	Left	40	2.5763	0.40026	0.06329	2.452252	2.700348
Superior joint space	Right	40	2.533	0.60349	0.09542	2.345977	2.720023
	Left	40	2.8093	0.57398	0.09075	2.63143	2.98717
Posterior joint space	Right	40	2.45	0.49578	0.07839	2.296356	2.603644
	Left	40	2.3953	0.41133	0.06504	2.267822	2.522778
Anteroposterior diameter of the condylar process	Right	40	7.617	1.13357	0.17923	7.265709	7.968291
	Left	40	7.7098	1.09365	0.17292	7.370877	8.048723
Mediolateral diameter of the condylar process	Right	40	20.639	1.95133	0.30853	20.03428	21.24372
	Left	40	20.6237	0.81461	0.1288	20.37125	20.87615
Angle between the condylar long axis & the mid-sagittal plane	Right	40	68.2075	8.78925	1.3897	65.48369	70.93131
	Left	40	68.19	9.17686	1.45099	65.34606	71.03394
Distance between the geometric center of the condylar process & the mid-sagittal plane	Right	40	50.8728	3.18183	0.50309	49.88674	51.85886
	Left	40	50.767	3.65846	0.57845	49.63324	51.90076
Anteroposterior difference between the right & left		40	0.5402	0.49843	0.07881	0.385	0.694

Comparison of the study parameters

Table 5 compares the parameters in the control group, showing no significant differences between the right and left sides for any parameter.

**Table 5:** Comparison of the study parameters in the Angle’s class II, division 1 (control) group t-test; p≤0.05 considered statistically significant

Parameter	Side	Sample size (N)	Mean	Standard deviation	Standard error mean	P value
Condylar fossa depth	Right	40	8.6335	0.78491	0.12411	0.12
	Left	40	8.4327	0.71958	0.11378	
Anterior joint space	Right	40	2.8713	0.47263	0.07473	0.095
	Left	40	2.7083	0.43156	0.06823	
Superior joint space	Right	40	2.5913	0.59117	0.09347	0.401
	Left	40	2.6648	0.62784	0.09927	
Posterior joint space	Right	40	2.5685	0.5547	0.08771	0.403
	Left	40	2.4565	0.61515	0.09726	
Anteroposterior diameter of the condylar process	Right	40	7.8915	1.0384	0.16419	0.596
	Left	40	7.9545	0.94533	0.14947	
Mediolateral diameter of the condylar process	Right	40	19.7812	1.0835	0.17132	0.255
	Left	40	20.1337	1.15207	0.18216	
Angle between the condylar long axis & the mid-sagittal plane	Right	40	65.475	3.84453	0.60787	0.769
	Left	40	64.855	8.41098	1.32989	
Distance between geometric center of the condylar process & the mid-sagittal plane	Right	40	50.9605	3.38447	0.53513	0.38
	Left	40	51.8608	3.06744	0.48501	

Table 6 highlights the comparison in the test group, where significant differences were noted in condylar fossa depth and superior joint space. The class I side had a significantly greater mean condylar fossa depth compared to the class II side. Furthermore, the superior joint space was significantly larger on the class II side compared to the class I side.

**Table 6:** Comparison of the study parameters in the Angle’s class II division 1 subdivision (test) group t test; p≤0.05 considered statistically significant; *denotes significance

Parameter	Side	Sample size (N)	Mean	Standard deviation	Standard error mean	P value
Condylar fossa depth	Class I	40	8.4565	0.76246	0.12056	<0.001*
	Class II	40	7.8527	0.89018	0.14075	
Anterior joint space	Class I	40	2.5863	0.51328	0.08116	0.918
	Class II	40	2.5763	0.40026	0.06329	
Superior joint space	Class I	40	2.533	0.60349	0.09542	0.006*
	Class II	40	2.8093	0.57398	0.09075	
Posterior joint space	Class I	40	2.45	0.49578	0.07839	0.492
	Class II	40	2.3953	0.41133	0.06504	
Anteroposterior diameter of condylar process	Class I	40	7.617	1.13357	0.17923	0.57
	Class II	40	7.7098	1.09365	0.17292	
Mediolateral diameter of condylar process	Class I	40	20.639	1.95133	0.30853	0.963
	Class II	40	20.6237	0.81461	0.1288	
Angle between the condylar long axis & the mid-sagittal plane	Class I	40	68.2075	8.78925	1.3897	0.992
	Class II	40	68.19	9.17686	1.45099	
Distance between the geometric center of condylar process & the mid-sagittal plane	Class I	40	50.8728	3.18183	0.50309	0.855
	Class II	40	50.767	3.65846	0.57845	

A comparison of the parameters between the control and test groups revealed significant differences in mean condylar fossa depth, anterior joint space, and mediolateral diameter of the condylar process (Table 7).

**Table 7:** Comparison of the study parameters between the control and test groups One way analysis of variance; p≤0.05 considered statistically significant; *denotes significance

Parameter	Side	Sample size (N)	Mean	Standard deviation	Standard error mean	P value
Condylar fossa depth	Control right	40	8.6335	0.78491	0.12411	<0.001*
	Control left	40	8.4327	0.71958	0.11378	
	Test class I	40	8.4565	0.76246	0.12056	
	Test class II	40	7.8527	0.89018	0.14075	
Anterior joint space	Control right	40	2.8713	0.47263	0.07473	0.014*
	Control left	40	2.7083	0.43156	0.06823	
	Test class I	40	2.5863	0.51328	0.08116	
	Test class II	40	2.5763	0.40026	0.06329	
Superior joint space	Control right	40	2.5913	0.59117	0.09347	0.195
	Control left	40	2.6648	0.62784	0.09927	
	Test class I	40	2.5330	0.60349	0.09542	
	Test class II	40	2.8093	0.57398	0.09075	
Posterior joint space	Control right	40	2.5685	0.55470	0.08771	0.514
	Control left	40	2.4565	0.61515	0.09726	
	Test class I	40	2.4500	0.49578	0.07839	
	Test class II	40	2.3953	0.41133	0.06504	
Anteroposterior diameter of the condylar process	Control right	40	7.8915	1.03840	0.16419	0.452
	Control left	40	7.9545	0.94533	0.14947	
	Test class I	40	7.6170	1.13357	0.17923	
	Test class II	40	7.7098	1.09365	0.17292	
Mediolateral diameter of the condylar process	Control right	40	19.7812	1.08350	0.17132	0.009*
	Control left	40	20.1337	1.15207	0.18216	
	Test class I	40	20.6390	1.95133	0.30853	
	Test class II	40	20.6237	0.81461	0.12880	
Angle between the condylar long axis & the mid-sagittal plane	Control right	40	65.4750	3.84453	0.60787	0.112
	Control left	40	64.8550	8.41098	1.32989	
	Test class I	40	68.2075	8.78925	1.38970	
	Test class II	40	68.1900	9.17686	1.45099	
Distance between the geometric center of the condylar process & the mid-sagittal plane	Control right	40	50.9605	3.38447	0.53513	0.436
	Control left	40	51.8608	3.06744	0.48501	
	Test class I	40	50.8728	3.18183	0.50309	
	Test class II	40	50.7670	3.65846	0.57845	

Multiple pairwise comparisons

Post hoc analysis showed that the class II side in the test group had significantly lower mean condylar fossa depth compared to both sides in the control group and the class I side in the test group (Table 8).

**Table 8:** Multiple pairwise comparisons of the study parameters between the control and test groups Tukey’s post hoc tests; p≤0.05 considered statistically significant; *denotes significance Reference group 1: control group right side; Reference group 2: control group left side; Reference group 3: test group class I side; Reference group 4: test group class II side

Parameter	Reference group	Control group	Mean difference	Standard error	P value	95% Confidence Interval	
						Lower Bound	Upper Bound
Condylar fossa depth	1	2	0.20075	0.17705	0.669	-0.2590	0.6605
		3	0.17700	0.17705	0.750	-0.2828	0.6368
		4	0.78075^*^	0.17705	0.001*	0.3210	1.2405
	2	3	-0.02375	0.17705	0.999	-0.4835	0.4360
		4	0.58000^*^	0.17705	0.007*	0.1202	1.0398
	3	4	0.60375^*^	0.17705	0.005*	0.1440	1.0635
Anterior joint space	1	2	0.16300	0.10206	0.383	-0.1020	0.4280
		3	0.28500^*^	0.10206	0.03*	0.0200	0.5500
		4	0.29500^*^	0.10206	0.023*	0.0300	0.5600
	2	3	0.12200	0.10206	0.631	-.1430	0.3870
		4	0.13200	0.10206	0.569	-0.1330	0.3970
	3	4	0.01000	0.10206	1.000	-0.2550	0.2750
Mediolateral diameter of condylar process	1	2	-0.35250	0.29522	0.632	-1.1192	0.4142
		3	-0.85775^*^	0.29522	0.022*	-1.6244	-0.0911
		4	-0.84250^*^	0.29522	0.025*	-1.6092	-0.0758
	2	3	-0.50525	0.29522	0.321	-1.2719	0.2614
		4	-0.49000	0.29522	0.349	-1.2567	0.2767
	3	4	0.01525	0.29522	1.000	-0.7514	0.7819

Intergroup comparison

Anteroposterior differences between the right and left sides in the control group and between the class I and class II sides in the test group, were not statistically significant (Table 9).

**Table 9:** Intergroup comparison of the anteroposterior difference between the right and left (control) and class I and class II (test) t test; p≤0.05 considered statistically significant

Group	Sample size (N)	Mean	Standard deviation	Standard error mean	P value
Angle’s class II, division 1	40	0.7385	0.54639	0.08639	0.094
Angle’s class II, division 1, subdivision	40	0.5402	0.49843	0.07881	

## Discussion

The strategic age criterion in the present study, reflected by the control group (23.67±3.56 years) and the test group (23.82±3.75 years), ensured that the participants had largely completed their facial growth and TMJ development. This research design aligns with a study by Akbulut et al. (2018), which evaluated condyle position in patients with Angle's class I, II, and III malocclusions [17]. Condyle-fossa relationships are critical in orthodontics, as they directly influence jaw movement and occlusal stability [18,19]. This study focused on individuals with mild to moderate convex facial profiles, characterized by an average to horizontal growth pattern and posterior divergence, resulting in a class II relationship. Additionally, subjects with a class II/end-on molar relationship, mild anterior protrusion, and minimal anterior crowding were also included. By examining individuals with these specific facial and dental characteristics, the study aimed to contribute valuable insights into the understanding and management of class II malocclusions, enhancing orthodontic practices and improving patient care. Rodrigues et al. (2007) utilized CBCT scans to investigate condylar symmetry and the condyle-fossa relationship in class II, division 1, and class III malocclusions [20]. The unique aspect of this study lies in its exploration of variations within the class II, division 1, subdivision, a subgroup that has received comparatively less attention in the existing literature.

Building on the foundational study by Rodrigues et al. (2007), which reported mean mandibular fossa depths of 7.98 mm on the right side and 8.25 mm on the left side [20], the present study offers essential reference points for understanding the natural variations in the mandibular fossa depth and contributes to the broader understanding of TMJ anatomy. In this study, the control group revealed no significant difference in condyle-fossa depth between the right (8.6 mm) and left (8.4 mm) sides. This suggests that symmetrical condyle-fossa depth is more pronounced in individuals without specific malocclusions. Conversely, the subdivision group displayed significant disparities between classes I and II. Notably, the mean condylar fossa depth was 8.4 mm on the class I side and 7.8 mm on the class II side, indicating that malocclusion may impact the structural features of the TMJ. This finding has important implications for orthodontic considerations, suggesting that malocclusions may influence both dental alignment and underlying skeletal morphology.

Malocclusions, or misalignments of the teeth and jaws, can induce adaptive changes in the TMJ spaces [2]. These adaptations manifest as variations in joint disc position, shifts in synovial fluid distribution, and adjustments in joint loading, all of which can affect the overall health and function of the TMJ [5]. In the context of this study, focused on Angle’s class II, division 1 malocclusion, the investigation delved into the specific dimensions of joint spaces - namely, the anterior, superior, and posterior spaces measured in the sagittal plane. Interestingly, in the control group with Angle’s class II, division 1 malocclusion, no significant differences were observed between the right and left joint spaces. However, in the test group with class II, division 1, subdivision malocclusion, significant disparities were noted in the superior joint space. Specifically, the superior joint space on the class II side was larger than on the class I side, indicating potential asymmetry or adaptive changes related to the malocclusion.

The study also provides further insights into the spatial dimensions of the mandibular condyles, such as the greatest anteroposterior and mediolateral diameters. Additionally, the angle between the long axis of the mandibular condyle and the mid-sagittal plane was evaluated, shedding light on angular relationships that contribute to the overall orientation of the condyles. A significant aspect of the study involved measuring the distance between the geometric centers of the condyles and the mid-sagittal plane. This distance, measured by a line passing through the geometric centers of the condyles and perpendicular to the mid-sagittal plane, was assessed for both sides. Rodrigues et al. (2007) previously reported significant differences in this distance in Angle’s class II, division 1 malocclusion [20].

Surprisingly, this study did not identify significant differences in the axial dimensions within both the control and test groups. However, a significant difference was found in the mediolateral diameter of the condyles when comparing the control group (Angle’s class II, division 1 malocclusion) to the test group. This suggests that while axial measurements may remain consistent within each malocclusion category, the mediolateral dimension displays distinct variations between class II, division 1 malocclusion and its subdivision. The outcomes of this study have broader implications, particularly in assessing condyle-fossa relationships in adults with class II, division 1 malocclusion and its subdivision. By examining all three dimensions of the condyle, this study provides a comprehensive evaluation of TMJ dynamics. Such understanding is crucial for refining diagnostic approaches and developing effective treatment strategies for individuals with these specific dental conditions.

A limitation of the study is that it predominantly included female subjects in both groups, which may introduce gender bias and limit the generalizability of the results.

Further studies could expand on this research by exploring the intricacies of the condyle-fossa relationship, including detailed analyses of their shapes and geometric positions across other malocclusion subdivisions. Additionally, functional aspects of the TMJ, such as range of motion, pain, or joint sounds, factors crucial for understanding the clinical implications of these findings, should be evaluated. Such in-depth investigations hold the potential to uncover unique patterns and variations specific to different malocclusion types, further illuminating the diverse anatomical and functional aspects involved.

## Conclusions

In cases of class II, division 1 malocclusion, there were no notable changes observed in the condyle-fossa relationship. The condyles maintained their concentric position within the fossae, and there was no significant difference in the dimensional or positional symmetry between the right and left condyles. However, in the class II, division 1 subdivision malocclusion, significant changes were detected. Specifically, the condyle-fossa depth was greater on the class I side compared to the class II side. Additionally, the superior joint space was larger on the class II side, indicating asymmetrical joint characteristics. For individuals with class II, division 1 malocclusion, noteworthy changes were identified, including increased condyle-fossa depth and variations in anterior joint space values. Moreover, in subdivision malocclusion cases, the larger mediolateral diameter of the condylar process was found.

## Appendices

**Table 10:** STROBE Statement—Checklist of items that are included in reports of cross-sectional studies (https://www.strobe-statement.org/checklists/)

	Item No	Recommendation
Title and abstract	1	(a) Indicate the study’s design with a commonly used term in the title or the abstract
		(b) Provide in the abstract an informative and balanced summary of what was done and what was found
Introduction		
Background/rationale	2	Explain the scientific background and rationale for the investigation being reported
Objectives	3	State specific objectives, including any prespecified hypotheses
Methods		
Study design	4	Present key elements of study design early in the paper
Setting	5	Describe the setting, locations, and relevant dates, including periods of recruitment, exposure, follow-up, and data collection
Participants	6	(a) Give the eligibility criteria, and the sources and methods of selection of participants
Variables	7	Clearly define all outcomes, exposures, predictors, potential confounders, and effect modifiers. Give diagnostic criteria, if applicable
Data sources/ measurement	8*	For each variable of interest, give sources of data and details of methods of assessment (measurement). Describe comparability of assessment methods if there is more than one group
Bias	9	Describe any efforts to address potential sources of bias
Study size	10	Explain how the study size was arrived at
Quantitative variables	11	Explain how quantitative variables were handled in the analyses. If applicable, describe which groupings were chosen and why
Statistical methods	12	(a) Describe all statistical methods, including those used to control for confounding
		(b) Describe any methods used to examine subgroups and interactions
		(c) Explain how missing data were addressed
		(d) If applicable, describe analytical methods taking account of sampling strategy
		(e) Describe any sensitivity analyses
Results		
Participants	13*	(a) Report numbers of individuals at each stage of study—eg numbers potentially eligible, examined for eligibility, confirmed eligible, included in the study, completing follow-up, and analysed
		(b) Give reasons for non-participation at each stage
		(c) Consider use of a flow diagram
Descriptive data	14*	(a) Give characteristics of study participants (eg demographic, clinical, social) and information on exposures and potential confounders
		(b) Indicate number of participants with missing data for each variable of interest
Outcome data	15*	Report numbers of outcome events or summary measures
Main results	16	(a) Give unadjusted estimates and, if applicable, confounder-adjusted estimates and their precision (eg, 95% confidence interval). Make clear which confounders were adjusted for and why they were included
		(b) Report category boundaries when continuous variables were categorized
		(c) If relevant, consider translating estimates of relative risk into absolute risk for a meaningful time period
Other analyses	17	Report other analyses done—eg analyses of subgroups and interactions, and sensitivity analyses
Discussion		
Key results	18	Summarise key results with reference to study objectives
Limitations	19	Discuss limitations of the study, taking into account sources of potential bias or imprecision. Discuss both direction and magnitude of any potential bias
Interpretation	20	Give a cautious overall interpretation of results considering objectives, limitations, multiplicity of analyses, results from similar studies, and other relevant evidence
Generalisability	21	Discuss the generalisability (external validity) of the study results
Other information		
Funding	22	Give the source of funding and the role of the funders for the present study and, if applicable, for the original study on which the present article is based
